# Infrared spectroscopy as a new approach for early fabry disease screening: a pilot study

**DOI:** 10.1186/s13023-024-03380-x

**Published:** 2024-10-10

**Authors:** Carolina Teles Barretto, Márcia Helena Cassago Nascimento, Bruna Ferro Brun, Tiago Barcelos da Silva, Pedro Augusto Costa Dias, Cassiano Augusto Braga Silva, Maneesh N. Singh, Francis L. Martin, Paulo Roberto Filgueiras, Wanderson Romão, Luciene Cristina Gastalho Campos, Valerio Garrone Barauna

**Affiliations:** 1 Postgraduate Program in Health Sciences, State University of Santa Cruz, Ilhéus, 45662-900 Bahia Brazil; 2 Chemometrics Laboratory of the Center of Competence in Petroleum Chemistry - NCQP, Federal University of Espírito Santo, Vitória, 29075-910 Espírito Santo Brazil; 3 Molecular Physiology Laboratory of Exercise Science, Federal University of Espírito Santo, Vitória, 29075-910 Espírito Santo Brazil; 4 Nephrology Department, Grupo CSB, Feira de Santana, Bahia, 44001-496 Brazil; 5 Biocel UK Ltd, Hull, HU10 6TS UK; 6 Chesterfield Royal Hospital, Chesterfield Road, Calow, Chesterfield, S44 5BL UK; 7 Department of Cellular Pathology, Blackpool Teaching Hospitals NHS Foundation Trust, Whinney Heys Road, Blackpool, FY3 8NR UK; 8 Federal Institute of Education, Science and Technology of Espírito Santo, Vila Velha, 29106-010 Espírito Santo Brazil; 9 Department of Physiological Science, Federal University of Espírito Santo (UFES), Av. Maruípe, 1468 – Maruípe, Vitória, Espírito Santo Brazil

**Keywords:** Alpha-galactosidase-A (α-Gal A) deficiency, Fabry disease, Infrared spectroscopy, Pattern recognition algorithms, PLS-DA

## Abstract

**Background:**

Fabry disease (FD) is a rare X-linked lysosomal storage disorder marked by alpha-galactosidase-A (α-Gal A) deficiency, caused by pathogenic mutations in the *GLA* gene, resulting in the accumulation of glycosphingolipids within lysosomes. The current screening test relies on measuring α-Gal A activity. However, this approach is limited to males. Infrared (IR) spectroscopy is a technique that can generate fingerprint spectra of a biofluid’s molecular composition and has been successfully applied to screen numerous diseases. Herein, we investigate the discriminating vibration profile of plasma chemical bonds in patients with FD using attenuated total reflection Fourier-transform IR (ATR-FTIR) spectroscopy.

**Results:**

The Fabry disease group (*n* = 47) and the healthy control group (*n* = 52) recruited were age-matched (39.2 ± 16.9 and 36.7 ± 10.9 years, respectively), and females were predominant in both groups (59.6% and 65.4%, respectively). All patients had the classic phenotype (100%), and no late-onset phenotype was detected. A generated partial least squares discriminant analysis (PLS-DA) classification model, independent of gender, allowed differentiation of samples from FD vs. control groups, reaching 100% sensitivity, specificity and accuracy.

**Conclusion:**

ATR-FTIR spectroscopy harnessed to pattern recognition algorithms can distinguish between FD patients and healthy control participants, offering the potential of a fast and inexpensive screening test.

**Supplementary Information:**

The online version contains supplementary material available at 10.1186/s13023-024-03380-x.

## Background

Fabry disease (FD, OMIM 301500)) is a rare X-linked lysosomal storage disorder marked by deficiency or absence of alpha-galactosidase-A (α-Gal-A) (EC 3.2.1.22; α-Gal A) lysosomal enzyme. This hydrolase deficiency, caused by pathogenic mutations in the *GLA* gene (NCBI reference sequence: NM_000169.3), leads to an accumulation of glycosphingolipids in biofluids and tissues, especially globotriaosylceramide (Gb3) and globotriaosylsphingosine (lyso-Gb3) [1]. The progressive storage of its nondegraded substrates results in multiorgan damage with life-threatening complications and reduces life expectancy [2]. FD has two main phenotypes referred to as “classic” or “late-onset”. In the male, the “classic” form, the signs and symptoms begin in childhood, including angiokeratoma, acroparesthesias, and hypohidrosis. In older age, patients develop mostly chronic kidney disease (CKD) and cardiac and cerebrovascular disorders. The “late-onset” form is characterized by residual enzyme activity and clinical manifestation confined to one organ, particularly the heart or kidney, in adulthood [3]. Females exhibit a wide range of clinical presentations attributed to the random inactivation of the X chromosome [4].

Due to the phenotypic heterogeneity and nonspecific clinical manifestations, the conclusive diagnosis of FD is challenging for physicians. Some diagnosis can be achieved in males through the measurement of α-Gal-A activity. In females, diagnosis is achieved exclusively through genetic testing since they often present with apparently normal enzymatic activity [5]. Furthermore, the finding of mutations never reported in the literature or variance of uncertain significance (VUS) contributes to difficulties in interpreting clinically relevant phenotypes [6].

Attenuated total reflection Fourier-transform infrared (ATR-FTIR) spectroscopy is a vibrational spectroscopy technique that captures the vibrational energy of organic molecules’ covalent bonds through IR radiation. The generated spectrum provides information about the sample’s chemical composition due to each molecule’s specific spectral molecular signature. Due to the complexity in discriminating different spectral datasets, chemometric methods have long been used to analyse such spectroscopic data from closely matched cohorts. Because of the vast amount of information rapidly acquired collected by the approach, feature selection and classification algorithms have been utilized to analyse biological datasets with high data complexity [7].

In 2022, Tolsik et al. applied another spectroscopy technique (Coherent anti-Stokes Raman, CARS) as a diagnostic tool to assess cardiac FD manifestation in an FD mouse model. In their study, the CARS measurements combined with multivariate data analysis allowed for differentiation between FD and control groups with 90–96% sensitivity. Indeed, CARS identified shifts in lipid/protein content between the two groups in cardiac tissue. This genotype differentiation was successful at a very early time point during disease development when only kidneys were visibly affected by Gb3 depositions [8].

ATR-FTIR spectroscopy has the advantage of being a fast and non-destructive technique that is reproducible, low-cost, requires a minimal volume of the biological sample, reagent free, and requires no previous sample handling [7]. This technique has been successfully tested as a tool for screening, diagnostics, and progression of diseases by examining several biofluid samples, including cells and tissue [9, 10]. Our group has previously shown the applicability of ATR-FTIR spectroscopy in biofluids, in both animal and human models, as screening for COVID-19 [11–13], iron overload [14], sepsis severity [15], and cancer [16]. Another metabolic genetic disease has also been studied (galactosaemia), and ATR-FTIR spectroscopy successfully discriminated among diabetic, healthy and galactosaemic subjects. IR spectroscopy performed well compared to the other tests and could easily be adapted for screening new-borns [17]. However, to the best of our knowledge, no studies have applied ATR-FTIR spectroscopy in FD patients. Therefore, this study aimed to evaluate the ATR-FTIR spectral dataset of plasma samples obtained from FD patients and healthy control participants to classify them through machine learning methods. We hypothesize that the spectra from these respective groups contain enough discriminating information to classify the samples, thus providing the potential for a fast, inexpensive screening test.

## Results

### Subjects characteristics

Table 1 Shows the characteristics of the volunteers included in the study. Females were predominant in both FD (59.6%) and HC (65.4%) groups. The ages ranged from 11 to 71 years (39.7 ± 16.9) in the FD group and 27 to 71 years (36.7 ± 10.9) in the HC group. Thus, there were no statistically significant differences between groups concerning gender and age (Table 1).

**Table 1:** Descriptive statistics by gender and age

	Fabry Patients	Healthy Control	*p*-value
Total *n*	47	52	
Male (*n*, %)	19 (40.4%)	18 (34.6%)	0.55^a^
Female (*n*, %)	28 (59.6%)	34 (65.4%)	
Age (years)			
Range	(11–71)	(22–71)	
Mean ± SD	39.7 ± 16.9	36.7 ± 10.9	0.30^b^

SD, standard deviation; ^a^Chi-Square; ^b^Unpaired T-test; test; *p* < 0.05

The clinical characteristics of the FD patients are summarized in Table 2 and the complete individual clinical data are described in Additional File 1. Regarding FD symptoms, men are more severely affected than women. Even within the same family, the clinical manifestations were heterogeneous among subjects. Only one patient, a young woman with an insertion and deletion mutation, had cerebrovascular involvement marked by the presence of a white matter lesions (WML) on MRI. The kidney was the most affected organ, reaching a percentage of 48.9%. More than 65% of the patients had been on enzyme replacement therapy (ERT) (Table 2), 84% of the men and 53% of the women in the FD goup. However, some patients on ERT indications were either not using medication due to treatment refusal or were newly diagnosed (Nos. 18, 26, 27, 44 and 47 in Additional File 1).

**Table 2:** Major clinical FD data

	*n* = 47
Angiokeratoma	13 (27.7%)
Acroparesthesis	36 (76.6%)
Hypo / Anidrose	23 (48.9%)
Cardiac involvement	3 (6.4%)
Kidney involvement	23 (48.9%)
Cerebrovascular involvement	1 (2.1%)
ERT	31 (65.9%)

We found seven different mutations distributed in 8 families (Additional File 2). All patients had the classic phenotype (100%), with a predominance of p.C52F and p.D264Y. No late-onset phenotype was detected. Two distinct families shared the same mutation (p.R227x), described as frequent in the literature [3]. Even though they do not know each other, a distant kinship relationship could not be ruled out since the families reside in the same state.

The mutation c.155G > T was not found in the Fabry databases. Nevertheless, Silva et al. reported it as a classic variant [18]. The mutations c.32delG insCCA and c.562del were not found in Fabry databases also; however, they are frameshift-type mutations, and patients present a classic disease phenotype (No. 45 and 47, respectively, in Additional File 1).

### ATR-FTIR spectral analysis

**Fig. 1:**
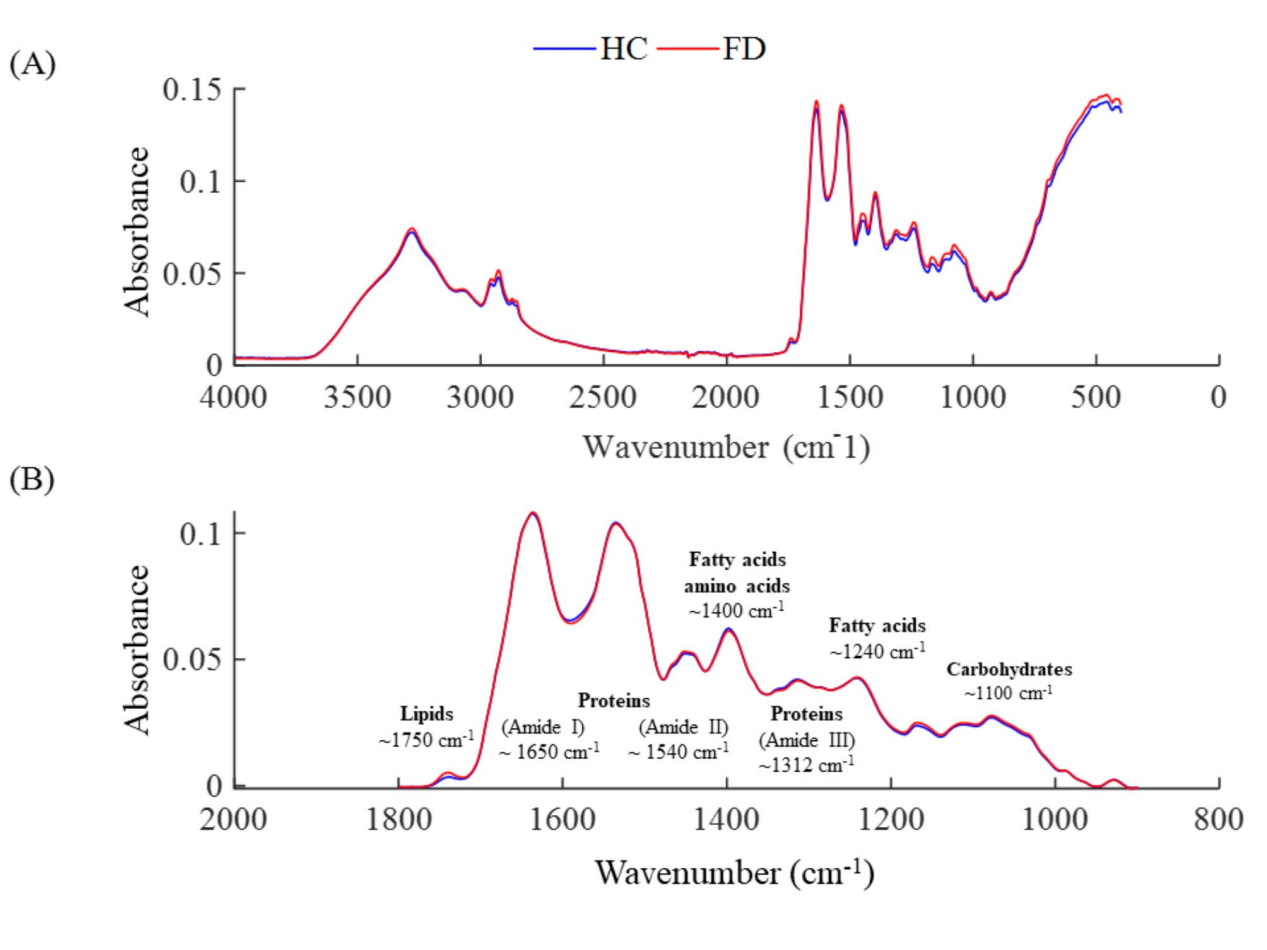
(**A**) Average raw spectra in the 4000 to 600 cm^-1^ region. (**B**) Average spectra after normalisation pre-processing in the 900 to 1800 cm^-1^ region with the position of biomolecular compounds

The average raw IR spectra and the normalized spectra truncated in the spectral fingerprint region of both groups are represented in Fig. 1A and B, respectively. Blue lines represent the HC group, and red lines represent the FD group. Organic compound assignments as biomolecule classes in biofluids are highlighted in Fig. 1B.

The Kennard Stone method was used to create the training and test sets for the partial least squares discriminant analysis (PLS-DA) model. A representative subset of samples was selected for the training set while a complementary subset was chosen for the test set. This method ensured that the original proportionality between the two classes was maintained, resulting in a more accurate and dependable model when applied to new data. The training set consisted of 69 samples while the test set consisted of 30 samples.

**Fig. 2:**
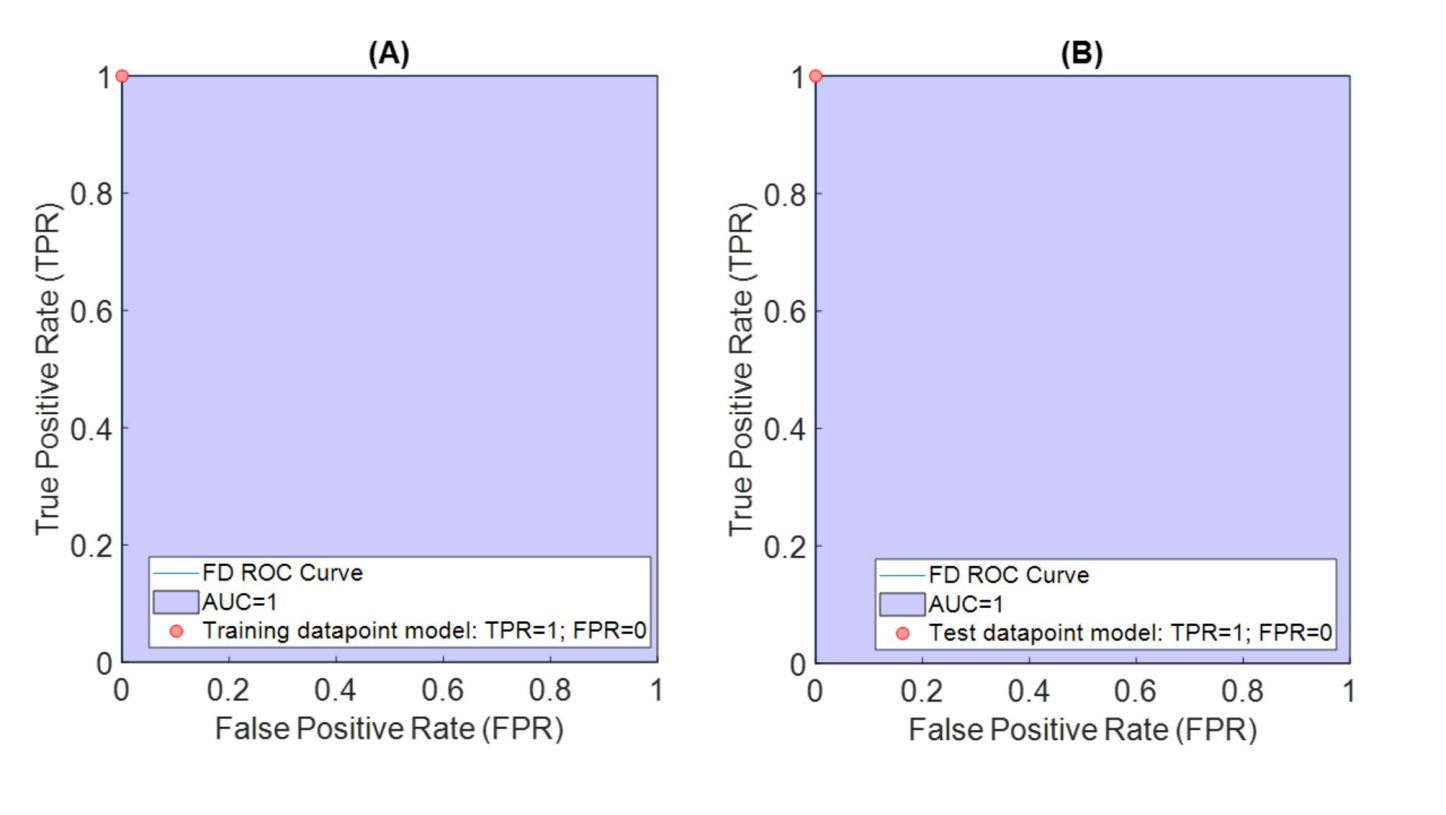
Receiver operating characteristic (ROC) curve made from the PLS-DA model. (**A**) Training ROC curve of the FD class, the area under the curve (AUC), and the training model point (TPR: true positive rate, FPR: false positive rate); (**B**) Test ROC curve of the FD class, the area under the curve (AUC), and the test model point (TPR: true positive rate, FPR: false positive rate)

Before modelling the PLS-DA, outliers were identified using the Q residual and T^2^ Hoteling control chart. Additionally, a cross-validation procedure was used to select 9 LVs, based on the cross-validation error rate. Table 3 shows the performance and confusion matrix of the PLS-DA model for the FD group. PLS-DA achieved 100% sensitivity and specificity in both the training and test subsets. Figure 2 demonstrates the receiver operating characteristics (ROC).

**Table 3:** Confusion matrix and performance characteristics for the fabry group of the training and test samples in the PLS-DA model

**Training**								
**Actual**	**Predicted**							
	Control	Fabry	**LV**	**Sens**	**Spec**	**Acc**	**PPV**	**NPV**
Control	36	0	9	100	100	100	100	100
Fabry	0	33						
**Test**								
**Actual**	**Predicted**							
	Control	Fabry	**LV**	**Sens**	**Spec**	**Acc**	**VPP**	**VPN**
Control	16	0	----	100	100	100	100	100
Fabry	0	14						

LV: latent variable; Sens: sensitivity; Spec: specificity; Acc: accuracy; PPV: positive predictive value; NPV: negative predictive value

**Fig. 3:**
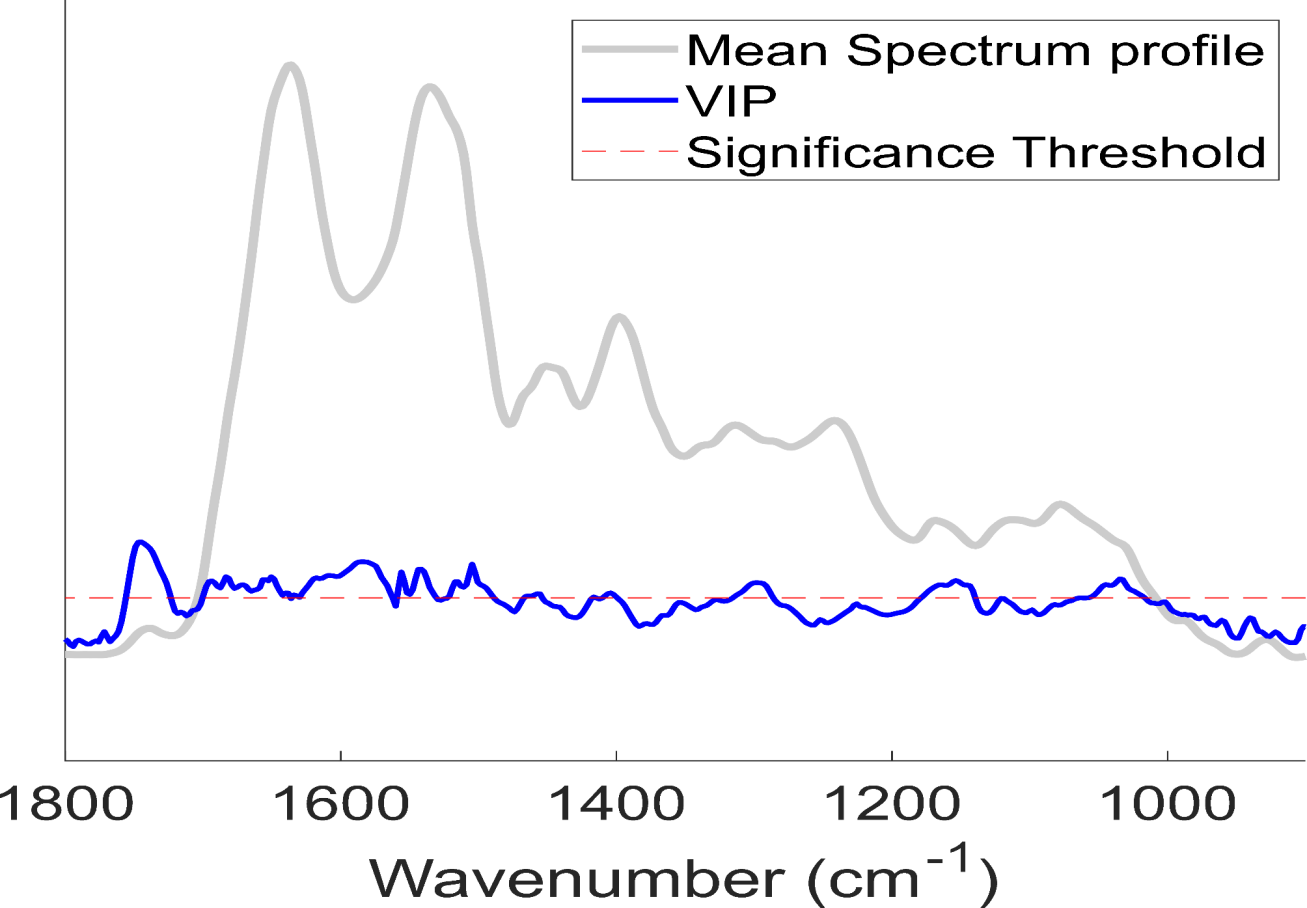
Variable importance in projection (VIP) score plot obtained in the PLS-DA analysis of the MIR spectra (900–1800 cm^-1^) dataset

Variable importance in projection (VIP) scores measure the variable’s importance in the PLS-DA model. It gives the main points contribution to the model. The VIP score plot obtained in the PLS-DA pointed to some spectrum ranges: 1020–1050 cm^-1^, 1140–1175 cm^-1^, 1287–1312 cm^-1^, 1490–1700 cm^-1^, and 1720–1755 cm^-1^ (Fig. 3). The greatest VIP index generated for the model was in the 1720–1755 cm^-1^ region, which corresponds to lipid assignments. These results agree with the physiopathology of FD, which is essentially characterized by the storage of glycosphingolipids.

## Discussion

FD affects 1 in 40,000 males and 1 in 117,000 live births [3], but newborn screening studies suggest an underdiagnosis and the numbers are higher than those reported [19, 20]. Furthermore, due to the phenotypic heterogeneity and nonspecific clinical manifestations, the suspicion of FD becomes a challenging diagnosis for physicians, and delays in diagnosis approximate 15 years [1]. Although enzyme measurement has been used as a screening tool in the active search for FD in high-risk male groups, these patients are mostly diagnosed at an advanced stage of the disease progression and often have irreversible organ damage. Although ERT can reduce organ injury and delay FD progression, the best therapy outcomes are achieved when initiated in early stages of the disease [21], corroborating the essential need for timely diagnosis.

In recent years, efforts have been made to detect affected FD patients. Lyso-Gb3 emerged as a sensitive biomarker and could assist in diagnosing heterozygosity, treatment decisions and monitoring [22]. Lyso-Gb3 also helps predict FD with relevant clinical manifestations, especially in new mutations with no defined pathogenesis [23, 24]. Although lyso-Gb3 is helpful, its levels must be interpreted with attention. In an experimental study, raised levels of lyso-Gb3, similar to those seen in FD patients, were found in plasma samples from patients with type 1 Gaucher disease [25]. Moreover, some heterozygotes have lyso-Gb3 levels similar to healthy individuals, with a remaining diagnostic gap in women with FD [23, 26]. Other lysosomal stress situations can induce local production of acid ceramidase from glycosphingolipids and generate sphingoid bases, suggesting that abnormal levels of lyso-Gb3 may be found in the circulation of individuals without the disease [27].

New approaches such as microRNAs [28], circular RNA [29], and metabolomics [30]  have been studied to expand our knowledge and assist in the management of FD, such as correlating genotype to phenotype, identifying the need to start and assess the effectiveness of enzyme replacement therapy (ERT). Jefferies et al. recently used machine learning to promote an algorithm to identify patients at high risk for FD. They used a broad dataset with confirmed FD patients (nearly 5000) and applied it to 1,000,000 patients in medical record data [31].

The vibrational spectroscopy technique provides important information about the biological samples analysed. ATR-FTIR spectroscopy is especially suitable for biofluid analysis because of the evanescence wave generated by the IR source in contrast to other IR techniques [7]. It efficiently detects subtle differences between the samples before significant tissue changes occur. FD biomarkers are limited to the current knowledge of biological disease pathways. The use of a molecular signature to screen for FD overcomes the limitations of unknowing biological pathways that could be involved in complex genetic diseases.

The VIP score plot obtained in the PLS-DA indicates the spectral regions that contribute significantly to the classification or discrimination between different groups. In our study, the main wavenumbers responsible for classification were: 1020–1050 cm^-1^ C-O stretching; 1140–1175 cm^-1^ C–O stretching of tyrosine; 1287–1312 cm^-1^ C–N stretching of Amide III; 1490–1700 cm^-1^ C = O and C–N stretching of Amide I and II; and 1720–1755 cm^-1^ C = O of esters, phospholipids and fatty acids [32].

The ATR-FTIR spectra include biological information in a complex matrix, which can be distinguished *via* multivariate analysis. Nevertheless, further studies are required to understand the specific biomarkers of the disease, which may include chromatographic and mass spectrometric techniques, because FTIR spectra can only provide clues about the functional groups associated with the disease and do not provide enough information to identify specific metabolites or molecular markers.

A computational algorithm capable of identifying a specific biomolecular signature for a disease with multifactorial clinical conditions has the potential to achieve greater sensitivity than a single biomarker. Our results showed excellent sensitivity in a single test for both sexes, simplifying screening in healthcare services for at-risk populations. It should be noted that although the numbers recruited for this study appear relatively small, FD is classed as a rare disease; in comparison with other studies in the literature to date, this is a large investigation into this condition.

Despite some women not presenting symptoms of the disease due to more significant inactivation of the mutant X chromosome, the presence of minimal biological alterations cannot be ruled out. Therefore, using a tool proved capable of detecting subtle differences between samples when associated with appropriate multivariate analysis methods. The absence of individuals with typical FD comorbidities in the control group may have been a limiting factor in the study. The impact of patients without FD, but with left ventricular hypertrophy and chronic kidney disease, for example, should be studied in the future. Another limitation was the predominance of patients with the p.C52F and p.D264Y mutations in the studied population. Therefore, new studies would also need to be carried out to include patients with the late-onset phenotype.

## Conclusion

Our study is the first to implement the vibrational spectroscopy technique in FD. The difficulty of FD diagnosis and the absence of a screening test, may be supported by the ATR-FTIR spectroscopy associated with multivariate data analysis. An algorithm was built to discriminate between plasma samples from patients with FD and healthy individuals, regardless of gender. Our promising results demonstrate the great potential of this approach in screening for FD, as it is a fast, inexpensive, and minimally invasive technique applicable to both sexes. However, while the results reported herein are promising, future research is required for proof-of-concept and application in an external cohort.

## Materials and methods

### Study population

The study was approved by the State University of Santa Cruz (UESC) Ethics Committee and was conducted according to the principles of the Helsinki Declaration. All subjects or their guardians signed a written informed consent form to collect blood samples and clinical data. The inclusion criteria were subjects with a genetic test of GLA gene pathogenic mutations. Between January 2021 and January 2022, 47 subjects were evaluated and diagnosed with FD from the Kidney Care Clinic and Cassiano Antonio Moraes University Hospital in BA and ES states of Brazil. The mutations were classified as coding for late-onset or classic phenotype, and it was based on the Fabry-Gen-Phen database (http://fabrygenphen.com/), OMIM database (http://www.ncbi.nlm.nih.gov/omim/) and/or the mutation database by H. Sakuraba (www.fabry-database.org). The clinical data, imaging examinations, and laboratory results were obtained from the patient’s medical records. The healthy control (HC) group consisted of 52 subjects matched by age and gender who were enrolled by informed consent.

### Sample preparation

Whole blood samples were collected into an EDTA tube and centrifuged at 1000 × g for 15 min. Plasma was aliquoted and conserved in 0.5 mL Eppendorf tubes at -80 °C. Until the day of the experiment, all samples were thawed overnight in the refrigerator. After preparing the sample, 10 µL was dropped onto an in-house-developed polished aluminium slide measuring 1 cm^2^ and left to dry for approximately 2 hours at room temperature before acquiring the spectrum. The experiments were performed in triplicate.

### ATR-FTIR spectroscopy

The equipment to process samples was Bruker Optics, Ettlingen, Germany, coupled with diamond crystal ATR, operated by OPUS 5.5 software, located at the Federal University of Espirito Santo (UFES). Spectra were acquired over a wavenumber range of 4,000–400 cm^− 1^, at a resolution of 4 cm^− 1^, with 32 spectra scans performed. The experiment was performed in triplicate, producing 156 spectra for the HC group and 141 for the FD group. A background spectrum was taken before every sample analysis to consider ambient room variations. The ATR crystal was cleaned with 70% alcohol and dried with absorbent paper between the samples analysed. The room temperature ranged between 20 and 24 °C, and the humidity ranged from 41 to 45%. The spectrum is delineated by band absorption, also called peaks, representing the functional groups’ vibrational modes. The location of the molecular functional groups in the spectrum is already well established in the literature [16, 32].

### Statistical analysis

The categorical variables are presented as proportions (percentage), and continuous variables are presented as the mean and standard deviation (SD). The chi-square test and T test were used for comparisons between groups as appropriate. A *p*-value < 0.05 was considered statistically significant. GraphPad Prism 8 was used for these statistical analyses.

Multivariate analysis was performed in MATLAB^®^ R2013a (The Mathworks, Natick, USA). First, the averaged spectra were truncated at the fingerprint region (1,800–900 cm^− 1^), following a baseline correction (airPLS algorithm) [33], smoothing with a Savitzky-Golay filter [34], and pre-processing with normalization by norm.

Partial least squares discriminant analysis (PLS-DA) was used as a supervised method to determine which class an unknown sample belongs to from the prior provision of known samples [35]. For this, we used the Kennard Stone algorithm [36] to split the pre-processed data into training (70%) and test (30%) subsets, ensuring representativeness and sample proportions between classes. Thus, the presence of outliers was evaluated from the Q residual and T^2^ Hoteling control charts. Cross-validation was performed by Monte Carlo cross-validation with 50 iterations in the training subset to select the optimum number of PLS components, called latent variables (LV). Finally, the built model was applied for validation by the test subset. This model was evaluated through quality performance parameters, such as sensitivity, specificity, accuracy, and the area under the curve (AUC).

## Electronic supplementary material

Below is the link to the electronic supplementary material.

Additional file 1: Patients’ characteristics and clinical data (This table contains the comprehensive clinical data of each individual in the patient’s cohort)

Additional file 2: Mutations characteristics (This table displays the characteristics of genetic mutations found in eight families that participated in this study)

## Data Availability

The data presented in this study are available on request. The data are not publicly available due to privacy restrictions.

## Abbreviations

α-Gal-A: Alpha-galactosidase-A
ATR: FTIR Attenuated total reflection Fourier-transform infrared
AUC: Area Under Curve
CKD: Chronic kidney disease
ERT: Enzyme replacement therapy
FD: Fabry disease
FPR: False Positive Rate
Gb3: Globotriaosylceramide
LV: Latent variables
Lyso-Gb3: Globotriaosylsphingosine
PLS-DA: Partial Least-Squares Discrimination Analysis
ROC: Receiver operating characteristic
TPR: True Positive Rate
VIP: Variable Importance in the Projection
VUS: Variance of uncertain significance
WML: White matter lesions
