# Supplementary material for: Infrared spectroscopy as a new approach for early fabry disease screening: a pilot study
Source: Orphanet J Rare Dis. 2024 Oct 10;19:373. doi: 10.1186/s13023-024-03380-x (PMC11466028; doi:10.1186/s13023-024-03380-x)
Supplement: Supplementary file 1 — Additional file 1: Patients’ characteristics and clinical data (This table contains the comprehensive clinical data of each individual in the patient’s cohort) [file 13023_2024_3380_MOESM1_ESM.docx]

| **Patient No.** | **Sex** | **Age (years)** | **GLA Mutation** | **Phenotype** | **ERT** | **Recombinant enzyme** | **Lyso-Gb3 (nmol/L)** | **Signals and  symptoms** |
| --- | --- | --- | --- | --- | --- | --- | --- | --- |
| 1 | F | 55 | c.155G>T | Classic | y | Agalsidase beta | N/A | Acroparesthesis |
| 2 | M | 29 | c.155G>T | Classic | y | Agalsidase beta | N/A | Acroparesthesis Angiokeratoma Hypohidrose Proteinuria |
| 3 | F | 32 | c.155G>T | Classic | n |  | N/A | Asymptomatic |
| 4 | M | 15 | c.155G>T | Classic | y | Agalsidase beta | N/A | Acroparesthesis  Angiokeratoma |
| 5 | F | 44 | c.155G>T | Classic | y | Agalsidase beta | N/A | Acroparesthesis |
| 6 | F | 64 | c.155G>T | Classic | n |  | N/A | Asymptomatic |
| 7 | M | 34 | c.155G>T | Classic | y | Agalsidase beta | N/A | Acroparesthesis Angiokeratoma Hypohidrose ESKD^a^ LVH |
| 8 | F | 16 | c.155G>T | Classic | n |  | N/A | Asymptomatic |
| 9 | F | 28 | c.155G>T | Classic | n |  | N/A | Asymptomatic |
| 10 | M | 16 | c.155G>T | Classic | y | Agalsidase beta | N/A | Acroparesthesis Angiokeratoma Hypohidrose Biopsy |
| *Continue...* | | | | | | | | |
| **Patient No.** | **Sex** | **Age (years)** | **GLA Mutation** | **Phenotype** | **ERT** | **Recombinant enzyme** | **Lyso-Gb3 (nmol/L)** | **Signals and  symptoms** |
| 11 | M | 18 | c.155G>T | Classic | y | Agalsidase beta | N/A | Acroparesthesis Angiokeratoma Hypohidrose Biopsy |
| 12 | F | 40 | c.155G>T | Classic | n |  | N/A | Asymptomatic |
| 13 | F | 36 | c.155G>T | Classic | n |  | N/A | Asymptomatic |
| 14 | F | 39 | c.155G>T | Classic | y | Agalsidase beta | N/A | Acroparesthesis |
| 15 | F | 31 | c.155G>T | Classic | n |  | N/A | Asymptomatic |
| 16 | F | 18 | c.155G>T | Classic | n |  | N/A | Asymptomatic |
| 17 | F | 71 | c.155G>T | Classic | n |  | N/A | Acroparesthesis |
| 18 | F | 40 | c.155G>T | Classic | n |  | N/A | Acroparesthesis Biopsy |
| 19 | F | 51 | c.790G>T | Classic | y | Agalsidase alfa | 6.1 | Acroparesthesis Hypohidrose |
| 20 | F | 41 | c.790G>T | Classic | y | Agalsidase alfa | 2 | Acroparesthesis Hypohidrose |
| 21 | F | 37 | c.790G>T | Classic | y | Agalsidase alfa | 3.3 | Acroparesthesis Microalbuminuria |
| 22 | F | 49 | c.790G>T | Classic | y | Agalsidase alfa | 6.5 | Acroparesthesis  Hypohidrose Microalbuminuria |
| 23 | F | 11 | c.790G>T | Classic | y | Agalsidase alfa | 1.7 | Acroparesthesis  Hypohidrose  Microalbuminuria |
| Continue... | | | | | | | | |
| **Patient No.** | **Sex** | **Age (years)** | **GLA Mutation** | **Phenotype** | **ERT** | **Recombinant enzyme** | **Lyso-Gb3 (nmol/L)** | **Signals and  symptoms** |
| 24 | M | 59 | c.790G>T | Classic | y | Agalsidase alfa | 7.4 | Acroparesthesis  Hypohidrose  Microalbuminuria |
| 25 | M | 66 | c.790G>T | Classic | y | Agalsidase alfa | 27.2 | Acroparesthesis  Hypohidrose  Microalbuminuria LVH |
| 26 | M | 11 | c.790G>T | Classic | n |  | 30.5 | Acroparesthesis  Hypohidrose  Microalbuminuria |
| 27 | M | 12 | c.790G>T | Classic | n |  | 37.2 | Acroparesthesis  Hypohidrose  Microalbuminuria |
| 28 | F | 61 | c.790G>T | Classic | y | Agalsidase alfa | 2.6 | Acroparesthesis  Hypohidrose  Proteinuria |
| 29 | F | 49 | c.790G>T | Classic | y | Agalsidase alfa | 3 | Acroparesthesis  Hypohidrose |
| 30 | F | 55 | c.790G>T | Classic | y | Agalsidase alfa | 2.5 | Acroparesthesis  Hypohidrose  Microalbuminuria |
| 31 | M | 55 | c.790G>T | Classic | y | Agalsidase alfa | 4.5 | Acroparesthesis  Hypohidrose |
| 32 | F | 41 | c.790G>T | Classic | y | Agalsidase alfa | 3.8 | Acroparesthesis  Hypohidrose  Microalbuminuria |
| *Continue...* | | | | | | | | |
| **Patient No.** | **Sex** | **Age (years)** | **GLA Mutation** | **Phenotype** | **ERT** | **Recombinant enzyme** | **Lyso-Gb3 (nmol/L)** | **Signals and  symptoms** |
| 33 | M | 55 | c.790G>T | Classic | y | Agalsidase alfa | 15.9 | Acroparesthesis  Hypohidrose |
| 34 | F | 47 | c.679C>T | Classic | n |  | N/A | Asymptomatic |
| 35 | M | 28 | c.679C>T | Classic | y | Agalsidase beta | N/A | Acroparesthesis Angiokeratoma ESKD^a^ |
| 36 | M | 20 | c.679C>T | Classic | y | Agalsidase beta | N/A | Acroparesthesis Angiokeratoma |
| 37 | M | 42 | c.679C>T | Classic | y | Agalsidase beta | N/A | Acroparesthesis Angiokeratoma ESKD^b^ |
| 38 | M | 43 | c.679C>T | Classic | y | Agalsidase beta | N/A | Acroparesthesis |
| 39 | F | 68 | c.679C>T | Classic | n |  | N/A | Asymptomatic |
| 40 | F | 64 | c.679C>T | Classic | y | Agalsidase beta | N/A | Acroparesthesis CKD |
| 41 | F | 46 | c.611G>A | Classic | y | Agalsidase beta | N/A | Acroparesthesis Biopsy |
| 42 | M | 50 | c.611G>A | Classic | y | Agalsidase beta | N/A | Acroparesthesis Angiokeratoma Hypohidrose ESKD^b^ |
| 43 | M | 20 | c.611G>A | Classic | y | Agalsidase beta | N/A | Acroparesthesis Angiokeratoma Hypohidrose |
| 44 | F | 64 | c.611G>A | Classic | n |  | N/A | Proteinuria |
| *Continue...* | | | | | | | | |
| **Patient No.** | **Sex** | **Age (years)** | **GLA Mutation** | **Phenotype** | **ERT** | **Recombinant enzyme** | **Lyso-Gb3 (nmol/L)** | **Signals and  symptoms** |
| 45 | F | 25 | c.32del Gins CCA | Classic | y | Agalsidase beta | 5.6 | Acroparesthesis  Angiokeratoma  Hypohidrose Cornea verticillata WML |
| 46 | M | 34 | c.801G>A | Classic | y | Agalsidase beta | N/A | Acroparesthesis Angiokeratoma Hypohidrose ESKD^b^ LVH |
| 47 | M | 35 | c.562del | Classic | n |  | 55.4 | Acroparesthesis  Angiokeratoma  Hypohidrose GI symptoms CKD |
