# Supplementary material for: Infrared spectroscopy as a new approach for early fabry disease screening: a pilot study
Source: Orphanet J Rare Dis. 2024 Oct 10;19:373. doi: 10.1186/s13023-024-03380-x (PMC11466028; doi:10.1186/s13023-024-03380-x)
Supplement: Supplementary file 2 — Additional file 2: Mutations characteristics (This table displays the characteristics of genetic mutations found in eight families that participated in this study) [file 13023_2024_3380_MOESM2_ESM.docx]

**Additional File 2 Mutations characteristics**

| **Location** | **GLA Mutation** | **Predictec Enzime Protein Change** | **Type** | **Phenotype** | **No. of patients with the mutation** | **No. of family** |
| --- | --- | --- | --- | --- | --- | --- |
| Exon 1 | c.155G>T | p.C52F | nonsense | classic | 18 | 1 |
| Exon 1 | c.32delG insCCA | unknown | del e ins | classic | 1 | 1 |
| Exon 4 | c.562del | p.(Ser188Profs*4) | frameshift | classic | 1 | 1 |
| Exon 4 | c.611G>A | p.W204x | nonsense | classic | 4 | 1 |
| Exon 5 | c.679C>T | p.R227X | nonsense | classic | 7 | 2 |
| Exon 5 | c.790G>T | p.D264Y | missense | classic | 15 | 1 |
| Exon 5 | c.801G>A | p.M267l | missense | classic | 1 | 1 |
